# Illustrations of the Diseases of the Breast

**Published:** 1829-04-01

**Authors:** 


ILLUSTRATIONS OF THE DISEASES OF THE BREAST. By *Sir Astley Cooper*, Bart. F.R.S. Sergeant-Surgeon to His Majesty—Consulting Surgeon of Guy's Hospital, &c. &c. In two Parts—Part I. Quarto, pp. 89, with nine beautifully-coloured Plates, containing numerous Figures. Longman and Co. Feb. 1829.

WHEN we recollect that hardly a ten-millionth part of the actual and useful experience of the profession is transmitted to posterity, or passes beyond the individual who first acquired it—when we reflect how very few indeed of those who honestly record what they know, have yet the gift of accurate perception, and the power of legitimate deduction, we may, or at least we should, be grateful to those eminent men in our profession, who, having passed a long period in unlimited practice, employ and amuse the leisure hours of mature age, in faithfully perpetuating the fruits of their observation for the benefit of their successors and of mankind at large. Such a man is Sir Astley Cooper. That the tide of fame and fortune which rolled upon him for a quarter of a century, till he was obliged to fly from it, should call up a moderate quantum of envy, is just as natural, nay, is as physically certain, as that the meridian sun should draw forth a halo of exhalations from a marshy soil. That Sir Astley Cooper is a pattern of infallibility—a genius of universal acquirement—a prodigy of learning—in short, an "admirable Creighton" of the age or of the profession, we are very far from asserting. There have been no such perfect characters of late, even among those who satirize the author of the volume before us. But we look upon Sir Astley as a man endowed with very good natural capacity—as possessing great zeal in the prosecution of surgical science—unequalled physical powers—assiduous observation—and what are better (in these days) than brilliant genius or superlative talent,—HONESTY and VERACITY! The clinical facts and practical precepts of Sir Astley Cooper, will be quoted and acted upon, long after the marble monument erected over his mortal remains shall have been mouldered into dust by the invisible waves of TIME, that "Edax rerum," beneath which all material fabrics must fall.

It is not generally known, but it will ultimately be made patent, that throughout a professional experience, unequalled in any age or country, the author of the volume before us has constantly employed most able artists to make accurate drawings of all important morbid structures which presented themselves to his view, or with the living symptoms of which he became acquainted—and this independently of preserving a great proportion of the said morbid structures in spirits. The consequence is, that Sir Astley Cooper possesses a collection of drawings infinitely surpassing that of any other surgeon in the profession. To make these available and useful to his brethren, through the medium of the press, is at once honourable to the individual, and beneficial to the public. We know not a more enviable occupation, in age, than that of perpetuating, for the good of posterity, the labours of youth. This task our author is now pursuing, while actually in the prime of life, and in the entire possession of every faculty:—in proof of which we shall, at once, proceed to an analytical delineation of the splendid volume before us—as far, at least, as the letter-press is concerned. We regret, indeed, that we cannot convey to our readers an adequate conception of the beauty and fidelity of the plates, which will only enrich the libraries of the more opulent members of the profession. We shall, however, endeavour to diffuse the practical descriptions and precepts of our experienced author over as wide a space as possible, through the instrumentality of the periodical press.

The work is dedicated, in a short but energetic address, to B. Harrison, Esq. Treasurer of Guy's Hospital;—and in the preface Sir A. informs us that he has divided these illustrations of mammary diseases into two parts—the malignant and non-malignant. It is with the latter class that the present volume is occupied. Our author thinks it right to observe, however, that although he has here confined himself to the description of diseases which are not malignant, and which do not arise from a vitiated state of the system, contaminating the parts in their neighbourhood, or even at a distance from their original seat, yet that—

> "Some of these swellings, when they have existed long in a dormant state, will have alterations produced in them by changes of the constitution, by which their extirpation may be rendered necessary; for malignancy may be lighted up in them by constitutional disease—by anxiety of mind—and by the cessation of the menstrual secretion."—*Preface.*

The work is divided into ten chapters, which we shall examine seriatim.

### Chap. I—Introductory.

In this chapter our author expatiates on the advantages which may be derived from the examination of morbid structures, and from a comparison of external symptoms with internal appearances.

Although we may not be able to cure some diseases, it is still a great advantage to be able to discriminate the remediable from the irremediable cases—the dangerous from the slight—those requiring the knife from those which do not demand so formidable a resource—and such as admit of a trifling operation from those that call for one of extreme severity.

The female breast is liable to almost all the diseases of other structures, besides some peculiar to itself. Yet, the uninformed surgeon is too apt to chime in with the vulgar opinion, and to confound all swellings of the mamma under the general term of Cancer. An examination of the diseased parts, after operations, shews the great variety which prevails in the nature and appearances of these swellings—and proves that, instead of their being all of one family, many genera of tumours actually exist. Some are the effect of acute, some of chronic inflammation—others of a specific action, malignant or harmless. It is therefore the surgeon's duty to discriminate these differences in the living body—which can only be done by a knowledge of what is revealed in the parts removed by operations, or the bodies of the dead. The following descriptive sketch is in Sir Astley's characteristic style, and is such a faithful copy from nature, that it will be instantly recognized as such, by every surgeon of the least experience.

"I have scarcely witnessed a stronger expression of delight than that which has illuminated the features of a female—perhaps the mother of a large family dependant upon her for protection, education, and support—who, upon consulting a surgeon for some tumour in her bosom, and expecting to hear from him a confirmation of the sentence she had pronounced upon herself, receives, on the contrary, an assurance that her apprehensions are unfounded. Pale and trembling, she enters the Surgeon's apartment, and baring her bosom, faintly articulates—Sir, I am come to consult you for a Cancer in my breast;—and when, after a careful examination, the Surgeon states, he has the pleasure of assuring her that the disease is not cancerous—that it has not the character of malignancy—that it is not dangerous, and will not require an operation; the sudden transition from apprehension to joy brightens her countenance with the smile of gratitude; and the happiness of the moment can hardly be exceeded, when she returns with delighted affection to the family, from which she had previously considered herself destined soon to be separated by death, with the alternative only of being saved by a dubious and painful operation." 4.

Our talented author divides mammary diseases into three classes—1mo. those resulting from common inflammation, acute or chronic—2ndo. complaints arising from a peculiar or specific action, but not malignant, or tending to contaminate contiguous structures—3tio. those diseases which are not only founded on local, malignant, and specific actions, but which are connected with a peculiar and unhealthy state of the constitution. By a malignant complaint, our author means a local diseased action, which not only affects the parts in which it is originally situated, but contaminates those contiguous. He considers it as resulting from a morbid state of the constitution, and it is frequently accompanied by a similar disease in other, and even remote parts of the body.

"The first of these classes comprehends the acute inflammation of the organ, as the milk abscess; the chronic inflammation, which remains for a length of time in a state of indolent swelling, and often terminates after a lapse of weeks or months in an indolent abscess; and, thirdly, a lacteal tumour, in which a chronic inflammation is followed by an obstruction in one of the lactiferous tubes, and produces a large lacteal or lactiferous swelling.

"In the second class of diseases of the breast we find several species of tumour, and they are as follow:—

"1st, the Hydatid;—2d, the Chronic Mammary Tumour;—3d, the Ossific;—4th, the Adipose;—5th, Large and pendulous Breast;—6th, the Scrofulous;—7th, the Irritable Breast;—8th, Ecchymosis of the Breast.

"In the third Class we find the two malignant diseases, which consist of the scirrhous and fungous tubercle." 5.

### CHAP. II.—EFFECTS OF COMMON INFLAMMATION IN THE BREAST.

The symptoms and treatment of acute inflammation in this organ do not differ materially from that in other structures, excepting in the severity of suffering which it produces.

"It is adhesive in the first stage, suppurative in the second, and ulcerative in the third. A firm and sensitive swelling of the whole or part of the mammary gland is produced in the first stage; and the dense cellular or fascial membrane with which it is enveloped, and by which all its parts are united, not easily yielding to the inflammatory swelling, often occasions most excessive suffering. The serous and fibrous portions of the blood are poured into, and fill the interstices of the inflamed structure, and the latter thus produces the solid swelling. To this enlargement succeeds a blush of inflammation upon the surface of the breast; throbbing, pulsatory, and very acute pain follows it; a particular prominence and smoothness are observed at one part of the tumour, with a sense of fluctuation from the presence of matter. The constitution is also highly irritated, which is evinced by the occurrence of shivering, succeeded by heat, and profuse perspiration. Over the most prominent part of the swelling the cuticle separates, ulceration follows in the cutis, and the matter becomes discharged through the aperture thus produced." 8.

The foregoing process requires from ten to twenty days for its completion. Sir A. considers the principal cause of this phlogosis to be the rush of blood to the breast, or "milk-draught," as it is called, when the infant is first put to the bosom. There are many other operative causes, however, as exposure to cold, the efforts of the child, the obstinacy of the nurse in not putting the child early to the breast,—and the too early introduction of stimulating liquors into the stomach of the mother.

"The best mode of treatment in these cases is to use, in the adhesive stage, a lotion of one ounce of spirit of wine, and five ounces of water, or of liquor plumbi dilutus to the part, and to purge the patient, by giving repeated doses of castor oil, or sulphate of magnesia. But if the patient suffer from the cold produced by the evaporation of the spirit, a simple tepid poultice may be substituted for it, occasionally applying leeches to the swelling, still recollecting that the chief dependence is upon purging." 9.

When matter is forming, poppy-fomentations and poultices must be employed—and anodynes should be given to mitigate pain. As to the question, should mammary abscess be opened or suffered to burst? the following is the reply. If the abscess be quick in its progress, anterior in its site, and unattended with severe sufferings, let it take its course. If the abscess be deep-seated—tedious—attended with great pain and much irritative fever, perspiration and insomnolency—discharge the matter by the lancet.—But we should not penetrate with this instrument through a thick covering of the abscess, as such an opening would not succeed in establishing a free discharge.

A quick succession of abscesses sometimes takes place in the breast, and leads to very protracted sufferings. Here opium and quinine will be required. Sometimes an abscess is produced at a great depth in the breast, and discharges itself by different apertures, forming sinuses of various extent.

"Now and then a deep-seated abscess forms between the posterior surface of the breast and the ribs, which, when it breaks, leaves a sinus which leads to the ribs. An exfoliation of part of the rib afterwards occurs, occasioning a very protracted suffering; and in these cases, as well as in the former, injecting the diluted acids is the best practice." 11.

These milk-abscesses are not always entirely devoid of danger. Sir A. once attended a lady of very delicate constitution, and who lay-in while under great anxiety of mind, in consequence of her husband being imprisoned. A milk abscess took place—discharged large quantities of matter—and then, instead of healing, the whole breast became excessively swollen—a true fungoid excrescence appeared—and this disease destroyed her life.

If the abscess be small, the child may be put to that breast as well as to the other;—but if the mamma be much involved in disease, the infant should be kept to the sound mamma, while the diseased one should be drawn by the nipple-glass.

"These abscesses are sometimes the result of soreness in the nipples, which appears in three forms:—first, in simple excoriation; secondly, in deep cracks at the junction of the nipple with the areola; and, thirdly, in deeper ulceration of the nipple itself, by which a part of it is removed. The suffering from these sores is often sufficiently great to prevent the frequent application of the child to that bosom, which leads to a great accumulation of milk, and to a degree of distention which occasions inflammation. To prevent this, the breast should be drawn; but the sooner the child can be restored to it, the better. The best application to the sore nipple is a solution of borax in water, in the proportion of a drachm of borax, three ounces and a half of water, and half an ounce of spirit of wine. Some use solutions of alum, some the sulphate of zinc, and some the supernatant liquor of a mixture of the liquor calcis with the submuriate of mercury. Also to prevent the nipples from becoming sore, to which many women are extremely subject, it is right to wash them some time before the lying-in with strong brine, which hardens the cuticle, and renders it less prone to ulceration and inflammation." 13.

### CHRONIC ABSCESSES.

The abscesses already described usually pass through their stadia in from three to five weeks; but, under chronic inflammation, an abscess is sometimes produced, which, from the slowness of its progress, and the absence of common inflammatory symptoms, is supposed to be a malignant tumour, and to require an operation.

"In proof of this, a woman was sent to me from Sussex who had a tumour in her breast, which I was requested to remove; and when she was seated before me for that purpose, I found, upon examining the swelling with attention, a fluctuation in its centre surrounded by a wall of hardness, with tenderness in the centre upon pressure. I therefore put a lancet into the seat of the fluctuation, to discover the nature of the fluid, and a considerable quantity of purulent matter was discharged through the orifice.

"I was also requested to see an out-patient at Guy's Hospital, who had a swelling in her breast with a fluctuation in its centre, which had existed several months, into which when a lancet was put, a large quantity of matter was discharged. Although there was no discoloration, and the swelling had existed several months, yet I thought it contained matter, from the sense of fluctuation, and from the tenderness the patient expressed upon slight pressure, which would not have been the result if a serous fluid had been collected.

" In similar cases I have seen the operation for removing the swelling begun, and in its progress the knife having accidentally entered the abscess, the Surgeon by escape of the matter having been informed of his error, the operation was suspended, and a poultice being applied, the case ended favourably." 15.

As, in these cases, there is generally some fault in constitution, or vitiation of the secretions, the compound calomel-pill should be given at night, and the bark with soda two or three times a day—or the compound infusion of gentian, with soda and rhubarb.

### Lacteal or Lactiferous Swelling.

This is so denominated by our author, because he supposes it to arise from a large collection of milk in one of the lactiferous tubes, the result of chronic inflammation in one of these conduits, near the nipple, with closure of its aperture, and obliteration of the canal for an inch or more.

" The patient applies to the Surgeon some time after delivery with a swelling in the breast; unpreceded by the symptoms of abscess, it distinctly fluctuates, and she complains exceedingly of a sense of distention in the part; and when the child is put to the breast to relieve it, the pain and distention are increased by the draught of milk which enters the breast so soon as the child begins to suck. The swelling is confined to one portion of the breast, from the nipple to the circumference of the organ, and it gives a distinct sense of fluctuation. The cutaneous veins are very large, but the part is otherwise undiscoloured. If a lancet be passed into the swelling, several ounces of milk are discharged; and the milk being suffered to rest for a few hours, forms a cream upon its surface. If a slight puncture only be made, the milk be discharged, and the opening suffered to immediately close, the accumulation recommences, and in a short time the same appearances and sufferings are renewed.

" When the distention of the swelling is excessive, it sometimes ulcerates, and discharges the milk which it has contained, by a small aperture at a little distance from the nipple; and the opening so produced often continues through the whole period of suckling, the milk being lost, from the aperture not being received into the child's mouth: and this opening is difficult to heal, until by weaning the child, and by purges, the secretion of milk be entirely checked.

" The treatment which this case requires is as follows:—If the mother be prevailed upon to wean her child, as the secretion of milk will soon cease in this obstructed part as in other parts of the breast, a simple puncture will suffice to relieve the distended tube of the milk which it contains.

" But if the child still continue at the breast, the opening may be made larger, and the milk be suffered to escape at the artificial aperture whilst the child is sucking; thus imitating the natural relief which the ulcerative process sometimes produces, until the secretion of milk ceases, from the weaning of the child, and from purges to the mother." 18.

### Chap. III. Hydatid Disease of the Breast.

There are four species of these swellings—three of which are non-malignant. To the description of *these* our author directs his attention.

1*st. Species.*—Simple bags containing a serous fluid, and which Sir A. calls *cellulous hydatids.* Symptoms :—The breast gradually swells, being free from pain or tenderness—becomes hard, without fluctuation—enlarges gradually for months or even years, sometimes acquiring great magnitude. Sir. A. has seen one breast weighing nine pounds.

" At first the swelling feels entirely solid, so that it bears a great resemblance to a simple chronic enlargement of the breast ; but after a great length of time, a fluctuation can at one part be discovered in it, and then the breast begins to increase more quickly; and in several parts similar fluctuations can be detected.

" The cutaneous veins become varicose ; but although the breast is immensely enlarged, it still continues almost entirely free from pain : but to this there are exceptions, for some persons feel an unusual heat, and some, as the breast increases, suffer pain in the part and in the shoulder.

" The tumour is extremely moveable upon the pectoral muscle ; is very pendulous ; and in some cases, the whole of the mammary gland, in others only a small portion of it, becomes involved in the disease.

" At length one of the fluctuating portions of the breast slowly inflames, ulcerates, and discharges a large quantity of serum, or of a fluid having its general character, but of a consistence somewhat more glairy; and the sac being emptied, and the external opening closed, if the fluid be entirely discharged, it is a long time before it re-accumulates ; and sometimes the sides of the sac adhere, and the cyst ceases to secrete. In other instances I have known the swelling break, and discharge a mucilaginous fluid mixed with serum ; and several of the cells in succession, and at distant periods, pass through the ulcerative process, and form sinuses, which are very difficult to heal." 22.

Except during ulceration, the general health remains undisturbed, and nothing but the apprehension of CANCER would induce the patient to make her case known or submit to an operation. However large the breast becomes, however extensively it ulcerates, however freely it discharges, yet the axillary glands remain free from disease.

*Dissection.* " When the swelling, and the breast in which it is situated, are examined, it is found, upon a careful dissection, that the interstices of the glandular structure itself, and the tendinous and cellular tissue connecting it, are in a great measure filled with fibrous matter, poured out by a peculiar species of chronic inflammation ; but in some of the interstices a bag is formed, into which a serous or glairy, or sometimes a mucous fluid is secreted, according to the degree of inflammation attending it; and this fluid, from its viscidity, and from the solid effusion which surrounds it, as well as from the cyst being a perfect bag, cannot escape into the surrounding tissues ; but by its quantity, its pressure, and by the gradual yielding of the bag, it becomes of very considerable size ; and vast numbers of these cysts are found to occupy each part of the breast, producing and supporting a continued but slow irritation, and occasioning an effusion of fibrous matter, by which the breast forms an immense tumour, consisting of solid and fluid matter. Within these bags of fluid, Hydatids, hanging by small stalks, but some, which from their appearance I supposed to be simple cells before I opened them, instead of being entirely hollow, had a cellular tissue within them, in which a fluid was collected, which, although it produced the appearance of cells or Hydatids on the outside, within assumed the character of anasarcous swellings.

" The breast, when not greatly enlarged, is almost entirely filled with cellulous Hydatids : some are produced in clusters, but the greater number are completely distinct from each other ; and in those cases in which the breast is but slightly increased, the constitution is but little irritable, and only a slight adhesive inflammation accompanies it." 23.

The size of these cells varies from that of a pin's head to that of a musket-ball, of which an example is seen in the first plate. The cyst containing the fluid is highly vascular, the veins being greatly dilated. This disease, in its first stage, resembles simple chronic inflammation; but is distinguishable from it by the absence of tenderness on pressure, and the good health of the patient —proving it to be an entirely local disease. In the second stage, when fluctuation exists, it may be distinguished by the distinct seats of the fluctuation, and by the absence of tenderness; but the surest criterion is puncture of the bag, when a clear serum will be evacuated, instead of pus. From scirrhous tubercle it is to be discriminated by the absence of those occasional acute darting pains that accompany malignant affection—by the continuance of health—and by the absence of that excessive hardness which characterises scirrhus. Sir A. has, however, seen a case "where true scirrhus had hydatids connected with it." The tumour was removed, and again returned. It had the usual pain of scirrhus.

"In the treatment of Hydatid disease, no local applications are beneficial, and the constitution requires no attention, because the general health does not suffer from the complaint.

"If only one bag is discovered, and that is of considerable size, it sometimes, if punctured, does not again fill, as will be seen in several of the cases.

"But when the enlargement is excessive—when a multitude of bags are produced—when the weight of the swelling becomes several pounds—when the breast is very pendulous, and drags upon the surrounding parts, and shakes upon every motion—when there is great apprehension, on the part of the patient, of some malignant disease, then the Surgeon will be wise in removing it.

"The operation itself is a simple piece of dissection, in which it is the best plan to secure each divided vessel in immediate succession, to prevent any great loss of blood; but it must be confessed that this is not absolutely necessary, as the operation does not require much time in its performance, and the vessels can be compressed by an assistant, whilst the Surgeon is removing the tumour; or, if he prefer it, each vessel may be secured in a ligature, as the operation proceeds.

"When the tumour requires removal for this disease, it is necessary to take away all the hardened and swollen parts of the breast, for they have cysts, or cells, formed in them; and if any cyst be suffered to remain, it will still continue to grow, and the remaining part of the breast to form an Hydatid tumour." 26.

It is a great consolation to know that this disease does not contaminate other structures in the neighbourhood. Numerous cases in illustration of the foregoing observations are detailed by our author; but of these we can only notice one or two.

Case VIII. Lady Hewett, Ætat. 60.

In November 1815 Lady H. fell against a chair, and some weeks afterwards felt uneasiness in the right breast, extending to the axilla. In January 1816, she discovered a small tumour in the same breast, just below the nipple. By the middle of the year the tumour was the size of a melon, and she was sent to Harrowgate, where she applied leeches every day for two months—and afterwards every second day till September. Pressure was next employed by means of bandages and machines, but without advantage. The swelling increased, and it was left to Nature till November 1817, when it began to undergo a change. It increased quickly and became soft at its upper part, apparently suppurating, but matter did not form, though poultices and fomentations were applied. An operation was then determined on, which our author performed on the 10th of June, 1818.

"The swelling was of great size, weighing nine pounds. It was in part solid; in some parts evidently contained a fluid; and upon the surface of the cyst part there was a slight blue tint. The swelling was moveable, and reached to the upper part of the abdomen. Lady H.'s general health was good.

"The first steps of the operation consisted in making a puncture into the tumour at its most prominent part, and discharging a quantity of serum from it; by which it was at once clear the disease was of the Hydatid kind, and the magnitude of the swelling was lessened.

"An incision was then made across the tumour, a little above its middle, and the flap of the integuments being raised, the upper part of the swelling was detached from the pectoral muscle, and with the handle of the knife the swelling was further separated; and a flap of skin being left below to meet that at the upper part, the operation was then concluded. Its removal was borne with great fortitude. Two arteries of considerable size required to be secured. The integuments were brought together by a single suture, and by adhesive plaster. On the 16th of June the wound was first dressed, and on the 30th Lady H. was quite well." 34.

We understand Lady Hewitt is now in good health at the age of 71. The removed tumour forms the subject of the second plate. A section is made through the tumour, and it is seen to be composed of a solid fibrous material, (coagulable lymph,) in which there are cavities containing hydatids. This is a splendid plate, and highly illustrative of the subject.

Case XII. Cellulous Hydatids with Scirrhous Tubercle.

This was an unfortunate case. The patient was a Miss S——, of Canterbury, aged 29 years, apparently healthy, but of thin and spare habit. Twelve months prior to consultation she perceived a small swelling in her left breast, attended with a sense of aching on pressure. When she had a cold on her, she experienced a thrilling pain, with darting in the part, and a sense of soreness in the nipple. The swelling increased, together with the pain and tenderness, and, on the 20th November, 1822, she came to London to undergo an operation. The tumour was now very hard, and impressed Sir Astley's mind with the idea of its being a scirrhous tubercle. Still her youth, health, and the fulness of the breasts, induced a hope that it might not be malignant. The operation was performed on Saturday, the 23d November. The tumour was deeply buried in the breast. On the succeeding Tuesday she had a rigor, succeeded by erysipelas, from which she narrowly escaped. Upon dissection of the tumour, it had the appearance of scirrhous tubercle at its upper part, while, at the lower, there were found several cellulous hydatids, as may be seen in the first plate. In about twelve months the disease returned, and the patient ultimately died of deeply ulcerated cancer, in the year 1826.

The second species of hydatid disease in the breast is of a very curious nature, and cannot be quite clearly understood without reference to the plate. The tumour was taken from the breast of Mrs. King, of Charing Cross, and the following graphical description we shall give in the words of the author.

"The breast was, in this case, enlarged, and in the greater part hardened by the effusion of fibrine (coagulable lymph) in lobes into the cellular tissue; but in several parts it contained bags of serum, and formed fluctuating cysts of various sizes. In each of these cells there hung a cluster of swellings, like polypi, supported by a small stalk; and the little pendulous projections appeared to float in the fluid which had been produced around them in the different cysts.

"Many Hydatids were found in a detached state, both in the fluid within the bags, and in the solid effusion in the breast; and taking the whole tumour, vast numbers of them had been formed in it.

"Their size varied, but the largest did not much exceed that of a barley-corn, the figure of which they assumed.

" In general they were of an oval form, or I ought to say oviform, as they were larger at one end than the other.

" When opened, they were found to be composed of numerous lamellæ, like the crystalline humour of the eye, or like the layers in the onion, which could be readily peeled from each other.

" When removed from the breast, they had a pearly appearance, and the laminated character of pearl internally.

" The cyst in which they were contained was a perfect bag, and it was composed internally of a membrane which was highly vascular, like other secreting surfaces; and the solid part surrounding the cyst had a greater number of vessels near the bag than at a remote distance from it; but the whole of the diseased structure was endowed with great vascularity, as will be seen in the Plate.

" Upon examining Plate the 4th, seven of these bags will be seen with clusters of pendulous tumours growing in them, connected by the stalks, which are delineated in Plate the 3d, and which contains sections from the same breast. Single Hydatids will be seen in the diseased solid structure, as well as cells containing a number of these bodies; and in one the cell is emptied, to shew its vascularity.

" It is doubtful if these structures are not of the nature of globular Hydatids, (which is the next I shall describe,) and which have perished from the pressure of the solid matter with which they are surrounded; or whether they are productions, or secretions of the arteries of the part: but the determination of this point must be left to future observation and diagnosis." 42.

In its external characters this disease resembles the first species described—the absence of tenderness common to both, will distinguish it from the simple chronic disease of the breast. From the former species of hydatid disease it cannot be discriminated. Extirpation is the only mode of relief; for no constitutional remedy can check the progress of the disease. A puncture of the cyst gives temporary relief—but the extirpation is free from danger, and by it the patient's mind is secured from future apprehension.

" Mrs. King, of Charing Cross, ætat. 58, had an enormous enlargement of her left breast, which she first discovered fourteen years ago, and then supposed it arose from a blow. When she first observed it, its size was that of a marble; it felt hard, and was unattended with pain.

" It appeared to be buried in the substance of the breast, and was not very moveable in the glandular structure. It increased gradually until two years ago, by which time it had acquired the size of a melon. At that period it seemed to increase suddenly, and to grow faster than before; but it was still unattended with pain, and her general health did not appear to suffer.

" Last Christmas it again suddenly increased; but was still devoid of any painful sensations, excepting that sometimes when she had a cold, she felt a slight uneasiness in the part.

" On the 30th of September, 1822, I first saw her, and the tumour then measured thirty-five inches in circumference; in the greater part it was solid, but in other parts it was soft and fluctuating and one bag evidently contained a large quantity of fluid.

" The solid portion of the tumour was placed at its upper part; the fluid occupied the lower part of the swelling. Her general health was good, but she suffered much from its weight drawing down the skin and pectoral muscle, and putting the nerves exceedingly upon the stretch.

" On the 1st of October I removed the tumour in the presence of Mr. Key, of Guy's Hospital, and Mr. Lavies, a Surgeon in Westminster.

" The large vessels, divided in the operation, were immediately secured, or compressed by an assistant as soon as divided, so as to prevent any loss of blood in the operation.

" The wound, when dressed on the seventh day, appeared healthy. The irritative fever consequent upon the operation was very slight, and she recovered without any untoward circumstances." 45.

### Third Species of Hydatid.

This is the animal or globular hydatid, consisting of a bag containing a fluid, having no vascular connexion with surrounding parts, and producing within its interior a multitude of bags similar to itself. There are few parts of the body in which hydatids have not been found, but the liver, ovaria, brain and cellular structure in the lower part of the abdomen, are the most frequent seats of this parasite animal.

In the human breast the hydatid is contained in a cyst, formed (so says our author) by the adhesive process—"for, wherever it is deposited, it excites irritation, and becomes surrounded and encased by an effusion of fibrine which is highly vascular, and its internal and secreting surface is directly applied to that of the hydatid, a slight moisture existing between them, they having no vascular connexion." In the mamma Sir Astley has only seen these hydatids exist singly, but in other parts of the body multiplied. The following anatomical notices accord with the observations of Rhudolphi, Laennec, and other modern anatomists.

"It has no opening or inlet, so that it must derive its nourishment by absorption from its external surface. It is composed of two coats; the external is of considerable density, and if any opaque body be placed behind it, it has the shining appearance of mother of pearl, and reflects the rays of light from its surface.

"It possesses a considerable share of elasticity, and rolls itself up when it is broken.

"This external layer is lined by a very delicate internal membrane, which appears to be its uterus; for from its interior a multitude of small Hydatids grow, which at first adhere to the membrane, but afterwards become detached, from its falling into the fluid which the Hydatid contains.

"If therefore the fluid contents of the Hydatid be collected in a glass, an immense number of small Hydatids will be discovered floating in them.

"Each of these small bags becomes in its turn a parent Hydatid, producing young upon its internal surface, in a similar manner to the parent cyst.

"I am induced to believe them to be distinct animals; first, because they have an existence and growth of their own, having no vascular connection with the part in which they are found, but being only incased and surrounded by a vascular and secreting cyst.

"Secondly, because they have the power of producing upon their interior surface their own species.

"Thirdly, that in the brain of sheep a similar bag is found, which, for several hours after the sheep has been killed, if it be put into warm water, has a distinct and very considerable vermicular motion; and, fourthly, because on the surface of the abdominal viscera, and sometimes in their interior, an Hydatid is found with a mouth and neck added to it; and consequently receives its food through the mouth, like other animals.

"The globular Hydatid, therefore, may be considered, as to its mode of nourishment, the link in the creation between the animal and vegetable, as it receives its nutriment by absorption as the vegetable does; but the tænia hydatigina, as it is called, which has a mouth, is a perfect animal, with respect to the manner of its nutrition.

"The Hydatid is supposed to be deposited in the structure in which it grows, carried there by the blood. Into whatever part it is thrown, it excites irritation, and becomes enclosed by an adhesive process, and which forms the cyst in which it is enveloped; but their origin is obscure, and the opinions respecting their deposition hypothetical.

"The parent hydatid is supported by a secretion from the internal surface of the cyst in which it is found; but the small Hydatids in it are probably nourished by the fluid which the parent Hydatid contains, so soon as they drop from, and cease to be connected with the parent cyst.

"When one of these hydatids is produced in the breast, an inflammation is excited by it, and a wall of fibrine surrounds it; it feels hard, and from the small size of the Hydatid a fluctuation cannot be discovered; but as the hydatid grows, although the quantity of solid matter increases, yet as the fluid in the Hydatid becomes more abundant, a fluctuation in the centre of the tumour may be ultimately perceived.

"Sometimes, when the Hydatid has considerably enlarged, it produces a suppurative inflammation; and when the matter is discharged, either by the lancet or by ulceration, the Hydatid escapes at the opening; and there is in the collection of preparations at St. Thomas's Hospital, an Hydatid which was thus discharged by ulceration from an abscess in the breast." 49.

The treatment of these hydatid tumours consists in making an incision into them, and discharging their contents, after which a simple poultice is sufficient to heal the wound. If the fluid re-accumulates, a seton must be passed into the bag, and the sac will slough. When the fluctuation escapes notice, and the surgeon removes the tumour, suspecting it to be of a scirrhous nature, then the hydatid is found, and the surgeon may confidently assure the patient that she is perfectly free from future danger. The distinguishing marks of this disease are its central fluctuation, its solid circumference, and the absence of tenderness on pressure. The disease is not dangerous prior to the operation, nor is this last followed by any bad consequences. There is a very good plate of the globular hydatid, furnished by the case of Mrs. Sarah Cornish, for which the author is indebted to Mr. Bayfield, Surgeon.

### CHAP. IV. CHRONIC MAMMARY TUMOUR.

This disease generally attacks young persons, from the age of 17 to 30 years, the constitution being generally healthy at the time, and during the progress of the disease. Sir Astley considers it as usually the result of sympathy with the uterus—"the excitement of the one organ leading to an increased determination and action in the other, and thus a new growth is produced." It occurs chiefly in single women, or in the married who have had no children.

"The symptoms which accompany this swelling are, that it grows from the surface of the breast rather than from its interior, and it therefore generally appears to be very superficial, excepting if it spring from the posterior surface of the breast, when it is deep-seated, and its peculiar features are less easily discriminated.

"It is an extremely moveable swelling, being chiefly attached by a portion of tendinous aponeurosis to the glandular structure of the breast, rather than buried within the gland; and therefore when moved, it glides over the surface of the breast.

"It begins without pain, and is therefore accidentally observed in the patient's ablutions; and it often continues for many years without exciting pain, or producing inconvenience; but in some cases it does become painful; the uneasy sensation extends to the shoulder, and the patient describes it to be of an aching or rheumatic kind.

"Generally it is not tender to the touch; but I have known it occasionally so, more especially before the patient is unwell at her monthly periods.

"Its growth is extremely slow, for I have removed one which had existed for five years, and which was not larger than a walnut; and I have seen another which had been growing seven years, and was but little larger than the former." 52.

On dissection, the disease is found to be contained in a bag formed of a similar fibro-tendinous structure to that which envelopes, as well as occupies the interstices of the glandular part of the breast;—and, in proportion to the magnitude of the tumour does this envelope become more and more distinct. It grows from the glandular structure of the breast, and remains connected with it by a thin process of a similar structure, which is lose and moveable. When laid bare, it appears to be composed of large lobes, like those of the breast; but when more completely unravelled, it is found to be composed of smaller and smaller lobes, similar in form, but differing in magnitude; and, after a short maceration in water the lobes are easily separated. The impression made on the mind during the dissection of the tumour is, that nature has formed an additional portion of breast, composed of similar lobes, but perhaps differing in structure by the absence of lactiferous tubes.

These tumours rarely acquire any considerable magnitude, weighing generally from one to four ounces. Mr. Bond, of Brighton, however, removed one which weighed a pound and a half, a size and weight which it acquired in two years. They are free from malignancy, and may exist many years stationary, disappearing after all. Upon a nice manipular examination of this tumour, it is found to be lobulated—that is, composed of a number of lobes connected together, but leaving depressions between them, and still preserving this conglomerate character, whatever size it may attain. The swelling might be termed the "lobulated mammary tumour." The general discriminating marks of this disease are as follow:—

"First, the youth of the patient: there are, however, some exceptions to this rule; but as the scirrhous tubercle is rarely seen under thirty years of age, so does this disease seldom happen after thirty.

"Secondly, the absence of pain; but this also is not constantly observed, although it is generally slight, and often the swelling exists many years without it.

"Thirdly, from the malignant diseases of the breast it is distinguished by the general health in this complaint remaining unaffected.

"Fourthly, the slow progress of the swelling, and the number of years it will exist in almost a stationary state.

"Fifthly, in its superficial situation upon the surface of the breast; for it is placed rather on the gland than in it.

"Sixthly, from its extreme mobility.

"Seventhly, above all, it is known from its lobulated feel; being distinctly composed of numerous lobes conglomerated into one mass, with a broken or divided surface.

"The cause of this disease is, as I have stated it to be, sympathetic with the uterus, and it arises from a great determination of blood to the part at certain times; but patients frequently ascribe it to a blow which they recollect to have received, or to the continued pressure of stays; and these circumstances of irritation may become the immediate exciting causes of the tumour, but the tendency to the disease is founded in uterine excitement.

"In the treatment of this complaint, it is right to learn if all the secretions be perfectly performed—if the liver secrete its proper quantity of bile—if the bowels be costive; but, above all, if the menstrual secretion be regularly performed, as regards its time, its quantity, its colour, and its duration.

"If the digestve functions are imperfectly performed, the Pil. Hyd. Sub. Comp. at night, and the Infus. Calumbæ cum Infus. Rhei. et Sodæ Carbon. twice in the day will be the best medicine; but if the uterine secretion be defective, the Pil. Hydrargyri gr. ij. Extr. Colocynthidis Compositi, gr. iij. Ft. Pilula, given every fourth or fifth night, with different preparations of Steel, to be taken two or three times per diem, will be the more appropriate constitutional remedies.

"As to local applications, one of the best is the Emp. Amm. cum Hydrargyro, if the diseased part be completely indolent; or the Iodine Ointment may be applied by friction upon the swelling, to excite the action of the absorbent vessels.

"But if there be heat or pain in the swelling, evaporating lotions, or simple poultices, are most productive of relief.

"It must be confessed, however, that these swellings are much out of the medical man's power to relieve, either by constitutional or local means; for as they are growths of long continuance, so will a great length of time be required to produce their absorption; and when they disappear, they seem to do so very gradually, from the cessation of that uterine excitement by which they have been produced, or by the part being called upon for its natural secretion of milk.

"But when the patient consults the Surgeon, she is very apprehensive of a cancerous or malignant disposition in the tumour; and he has the power of relieving her mind by the following declarations, which time will verify.

"First, that the disease is decidedly not malignant; and therefore if it do not yield to treatment, it is not dangerous to life.

"Secondly, that it does not absolutely require an operation; for it will continue for years, and then gradually disappear.

"Thirdly, if the patient be anxious to have the malady removed, from an apprehension of its becoming malignant, and if she determine to have it done, the operation is of the simplest kind; and it is not followed by any serious symptom, immediately or remotely; nor is the disease liable to recur." 57.

Single women having this disease are very apt to inquire of the surgeon if they may marry. Sir Astley's reply has always been, that marriage would be beneficial. This answer may also be given to the future husband, if consulted by him, which is sometimes the case. The disease often disappears during lactation, and does not prevent pregnancy. Numerous cases are detailed by our author, in illustration of the description, pathology, and surgical anatomy here laid down, and for which we must refer to the work.

### Chap. V.—The Cartilaginous and Ossific Tumour.

In chronic and specific inflammations of the mamma, a gelatine is sometimes effused, resembling that which supplies the bones in the fœtus, and parts of the bones in infants. This gelatine becomes vascular—ultimately resembles cartilage—and forms a nidus for bone. One case is detailed in illustration of this disease, of which a plate is also given. The following is the case.

"Mary Farmer, aged 32 years, applied to me for a swelling in her breast, which she had observed for fourteen years.

"The pain in it was very severe; the skin which covered it felt very warm when compared with the surrounding parts; and it required the constant application of evaporating lotions to moderate its warmth. The tumour was excessively hard, very painful before menstruation, but greatly relieved after it.

"Various applications were tried, *viz.* fomentations, poultices, and stimulating plaisters, but they neither disposed it to absorption nor to suppuration; and as all the means employed to disperse it were quite unavailing, she was anxious for its removal.

"The glands in the axilla being free from disease, as the complaint had existed for so long a period, and her general health seemed to be perfectly good, I recommended the operation, as affording the only hope of cure.

"Upon examination of the swelling after its removal, the larger portion of it had the appearance of that cartilage which supplies the place of bone in the young subject: the remaining part was ossific." 65.

### Chap. VI.—Adipose Tumour.

Sir Astley has had two occasions to operate for the removal of these unwieldy masses. In the first case, the tumour began at the posterior part of the breast, and grew between the gland and the surface of the pectoral muscle. In the second, all those lobes of fat, which are interspersed between the different portions of the mammary gland, became enlarged, and formed a swelling which, prior to incision being made, seemed to involve the whole of the glandular structure of the breast. But when the operation was performed, the different lobes of adeps which formed the tumour, could be drawn away from the gland itself. One of these cases we shall give in the original.

"Mrs. Smith, of Great Yarmouth, Norfolk, was admited into Guy's Hospital in August 1805, for an enormous tumour in the left breast: its circumference was thirty-one inches, and its length ten inches and a half. It was removed August the 29th, by making, first, a semicircular incision at the anterior and upper part of the tumour, and then drawing down the swelling; an incision was made along its upper part until the pectoralis muscle was exposed, from which the tumour was afterwards dissected from the upper to the lower part, its own weight drawing the cellular membrane to great extent, so as to render its detachment easy. As the different vessels which supplied it were cut through, the fingers of an assistant were applied upon them, and very little blood was lost in the operation; but in order to complete it, a very large portion of integument, the whole of the breast, and the tumour which was situated behind it, were removed. Several sutures were used to approximate the edges of the skin, which were also brought together by means of adhesive plaister; and the patient soon recovered.

"The tumour, which is preserved in the collection at St. Thomas's Hospital, weighed 14 lbs. 10 ounces." 67.

### Chap. VII.—Large and Pendulous Breast.

The glandular structure of the mamma sometimes grows to an enormous size, becoming extremely pendulous, so as to reach the fore part of the abdomen. This is not the effect of relaxation—but an absolute growth of the secreting lobes, enlarged, and occasionally very tender. The most remarkable case of this kind was sent to Dr. Babington, and our author, from Pembrokeshire, the young lady handing to Sir A. the following letter.

"Sir,

"I am induced to request your advice in the case of Miss ———, who about three years ago was first affected with an enlargement of the left mamma, which continued increasing; and the right breast then began also to enlarge, until they attained their present dimensions. She is now fifteen years of age, and of good general health: the catamenia appeared about twelve months ago. I was requested to see her last winter, in company with Mr. Gregory, of Milford, and she has taken various emmenagogue medicines and gentle laxatives; and she was enjoined regular exercise and sea-bathing. The catamenia returned at three or four regular intervals, at which time the mammæ considerably decreased in size; but since May last, the periods have been very distant, and the discharge is very small in quantity.

"The mammæ are now of extraordinary dimensions. The circumference of the left is twenty-three inches and a half, that of the right is twenty-two inches, and they are pendant like a pear, as the neck is comparatively narrow. I cannot perceive any tumour, either in the breast or in the axilla. The skin feels and appears to be natural. Her appetite is good, and the bowels are kept regular by occasional doses of neutral salts. She suffers no pain whatever in either mamma, but she does not appear so lively as girls of her age, but indeed, on the contrary, is heavy and dull. In other respects there is nothing peculiar in this young lady's case.

"I am, Sir, your obedient servant,

"W. D. Jones.

"The local treatment of this case consists in the application of a suspensory bandage from the back of the neck, under each breast, to produce artificial support; and the principle which is to be observed in the constitutional treatment of this malady, is to increase and to support the menstrual secretion; and for this purpose the exhibition of different forms of Steel united with Aloes, will be found the most efficacious medicine.

"The Ferrum Ammoniatum—the Mistura Ferri Composita—the Carbonate of Iron, will be the forms of Steel which, united with Aloes, will be most beneficial; and if the biliary secretions be defective, the Pil. Hyd. Sub. Comp., or the Hyd. cum Creta, will be the best medicines." 71.

Women who have led a life of celibacy to the age of 30 or 35 years, whose menstrual secretion has become very defective, and who are subject to severe fluor albus, are liable to have their breasts enlarged, not pendulous, each lobe of the gland being distinctly felt, moving freely, one on the other. Both breasts are affected; but one generally more than the other, accompanied with occasional pain, especially at the menstrual period, the catamenia being slight, pale, and of short duration. The breast, after being sometime enlarged, begins to waste; and, in a few years, it is in a great degree, absorbed. The treatment consists in restoring, if possible, the menstrual secretion by the means already alluded to, and by the use of the warm hip-bath—by the local application of leeches where there is pain, and by the emplastrum ammon. cum hydrarg.

### Chap. VIII.—Scrofulous Swelling of the Breast.

In young women who have enlargement of the cervical glands, scrofulous tumours sometimes, though rarely, form in the breasts, unattended by pain—are distinctly circumscribed—very smooth on the surface—and scarcely tender on pressure. They are indolent, varying with the state of the constitution, diminishing as it improves, and increasing as it degenerates. They can only be discriminated from the simple chronic inflammation of the breast by the absence of tenderness, and by the existence of other diseases of a similar kind in the absorbent glands of other parts of the body. They are unattended with danger, never degenerating into malignancy. They require no operation—though our author has seen amputation performed from ignorance of the true nature of the disease.

"The treatment in this case consists in improving the constitution by a warm and dry atmosphere—by an equally regulated temperature—by tepid sea-bathing—by gentle and regular exercise—by animal food of the most digestible kind—by milk—and by a farinaceous diet—a diet which shall nourish without exciting feverish heat, or calling much upon the powers of digestion.

"The best medicines are Carbonate of Iron and Rhubarb; the Hyd. cum Creta with Rhubarb; a grain of blue Pill, and two or three grains of Quinine; Infusion of Calumba with Rhubarb and Soda; for I conclude it will be admitted by every one who deserves the title of a Surgeon, that we possess no specific remedy for this disease, but that we are required to assist the digestive powers, make better blood, and convey it to the system by an increased vigour of the constitution.

"Local treatment avails but little: a stimulating plaister or a lotion to the tumour, when the health is improved, may excite the absorbents to remove it." 75.

### Chap. IX.—Irritable Tumour of the Breast.

The mamma is liable to become irritable without any perceptible swelling, as well as to form an irritable tumour, composed of a structure unlike that of the gland itself, and apparently of a specific growth. In the great majority of instances it occurs between the age of 16 and 30 years; and never, as far as our author has observed, before the age of puberty.

"When the complaint affects the glandular structure of the breast, there is scarcely any perceptible swelling, but one or more of its lobes becomes exquisitely tender to the touch; and if it be handled, the pain sometimes continues for several hours. The uneasy sensation is not confined to the breast alone, but it extends to the shoulder and axilla, to the inner side of the elbow, and to the fingers; it also affects that side of the body even to the hip; the patients cannot sleep on that side, and the pain is sometimes so severe as to prevent even their resting on the diseased side; and the weight of the breast in bed in some instances occasions intolerable pain.

"Patients also state that heat and cold frequently succeed each other in the breast; and it would seem the pain resembles that in the Tic-douloureux, darting like electricity through the part, and through the neighbouring nerves. When the pain is most severe, the stomach sympathizes, and vomiting is produced. The suffering is very much increased prior to menstruation; is somewhat relieved during the period, and decreased after its cessation. There is no external mark of inflammation, as the skin remains undiscoloured.

"In some cases only a small portion of one breast is affected; in others the whole, and not unfrequently both of the breasts.

"This painful state remains for months, and even for years, with little intermission; but it has no malignant tendency: and an operation, where there is no distinct tumour, must be entirely out of contemplation.

"Besides this irritable and painful state of a whole, or part of the breast, a tumour sometimes is found distinctly circumscribed—highly sensitive to the touch—acutely painful at intervals, more especially prior to menstruation—very moveable—often not larger than a pea, seldom exceeding the size of a marble: generally one only exists, but in other cases there are several similar swellings." 78.

Although they continue for years, they vary but little in size. Sir A. has never seen them suppurate—they sometimes disappear spontaneously. Upon dissection they are found to consist of a solid and semi-transparent substance, with fibres interwoven in it, but without any regular distribution. He has not been able to trace any filament of nerve into them. They seem to be productions of the cellular membrane of the part rather than of the glandular structure. The diagnosis is not difficult. The pain—the tenderness to the slightest touch—the suffering which succeeds examination; these distinguish it from the hydatid, the chronic mammary tumour, and the scirrhous and fungous tubercle. The disease is met with in persons of an irritable and nervous temperament, in whom there is excessive irritability of the system, accompanied with diminished power. The menstrual secretion is generally very deficient—in a few cases morbidly copious—very rarely perfectly regular. The fluor albus is a frequent concomitant of this complaint. The patient generally traces the disease to some blow, or injury from pressure on the part. The treatment consists in lessening the irritability of the system—in lulling the local suffering—and in restoring the defective or diminished menstruation. The best local remedy is the application of a plaister composed of equal parts of soap cerate and extract of belladonna; or a poultice with solution of belladonna and bread. Oil-silk worn on the breast, or a hare-skin, or some other fur, by exciting perspiration, aids in soothing and tranquillising the part. Leeches may be applied when the pain is excessive, but if too frequently used, they induce debility, and increase irritability. As constitutional remedies, calomel, opium, and conium should be given for a time, with an occasional aperient, and then the following is recommended by our author.

℞ Extr. Conii.
—— Pap. āā. gr. ij.
—— Stramonii. (e Semin.) gr. ss.
Misce fiat pilula bis terve in die sumenda.

The half grain of stramonium is sometimes too strong, and may be diminished. To restore the uterine secretion, Sir A. recommends the carbonas ferri, the ferrum ammoniatum, or the mistura ferri composita, combined with aloes. The hip-bath at 100 or 105° is beneficial. No operation is necessary.

### CHAP. X.—ECCHYMOSIS OF THE BREAST.

Allied to the irritable breast is a bruised appearance in this organ, occurring at each menstrual period, and accompanied with exquisite sensibility, pain, and tenderness.

"The symptoms of this complaint are as follow:—It occurs in girls who are in most instances under twenty-two years of age. It is preceded by severe pain in the breast and arm. The extravasation of blood begins a few days before menstruation, and it appears principally in a large spot, as if a severe blow had been inflicted. Smaller and less vivid spots may also be observed in other parts of the breast: it is sometimes a concomitant of an unusually large bosom. The part is exquisitely tender to the touch, and the pain with which it is accompanied, passes down along the inner side of the arm to the ends of the fingers. It disappears a week after menstruation, in some cases; but in others, when it is more severe, it continues until the next time the patient is unwell. It looks like the ecchymosis which often succeeds the application of leeches; or like the extravasation of blood under the skin, which occurs in the arm after bleeding, when the opening in the skin has been smaller than that in the vein.

"It is a curious occurrence, strikingly shewing the strong sympathy which subsists between the uterus and breast; for it is evidently the effect of the great determination of blood to the bosom just prior to the period of menstruation; and it indicates excessive irritability of the constitution, as well as the great delicacy and debility of the blood-vessels, which are unable to support this sudden determination which such sympathy produces.

"This complaint is entirely unattended with danger; but being accompanied with diminished, irregular, and sometimes profuse uterine secretion, and by considerable debility and irritability of the constitution, two objects must be kept in view in its treatment:—the one is, by different forms of Steel medicines, to increase the quantity, and render regular the menstrual discharge; and the other, to augment the strength of the system, by the Infusion of Roses with Sulphate of Quinine.

"As to local treatment, the best application is the Liquor Ammoniæ Acetatis, with Spirits of Wine, in the proportion of five ounces of the former, and one of the latter."

Several cases are detailed in illustration, but these we need not dwell on.

We have now exhibited a very full analysis of the letter-press of this valuable volume; but the plates constitute, of course, the most important portion of the work, and are admirably executed. Every surgeon of respectability in the profession will place these plates in his library, for constant reference, when the subject of mammary diseases is under his consideration. In respect to the description, pathology, and didactic precepts contained in the letter-press, we hope we have done some service to the great mass of the profession by wide circulation of them in every direction. We shall look with great interest to the second part of the work, embracing cancer and other malignant diseases of the human mamma.

